# The Phenolic Profile and Anti-Inflammatory Effect of Ethanolic Extract of Polish Propolis on Activated Human Gingival Fibroblasts-1 Cell Line

**DOI:** 10.3390/molecules28227477

**Published:** 2023-11-08

**Authors:** Anna Kurek-Górecka, Małgorzata Kłósek, Grażyna Pietsz, Zenon P. Czuba, Sevgi Kolayli, Zehra Can, Radosław Balwierz, Paweł Olczyk

**Affiliations:** 1 Department of Community Pharmacy, Faculty of Pharmaceutical Sciences in Sosnowiec, Medical University of Silesia in Katowice, Kasztanowa 3, 41-200 Sosnowiec, Poland; polczyk@sum.edu.pl; 2 Department of Microbiology and Immunology, Faculty of Medical Sciences in Zabrze, Medical University of Silesia in Katowice, Jordana 19, 41-808 Zabrze, Poland; mklosek@sum.edu.pl (M.K.); gpietsz@sum.edu.pl (G.P.); 3 Department of Chemistry, Faculty of Science, Karadeniz Technique University, 61080 Trabzon, Turkey; skolayli61@yahoo.com; 4 Department of Emergency Aid and Disaster Management, Faculty of Applied Sciences, Bayburt University, 69000 Bayburt, Turkey; zehracan61@gmail.com; 5 Institute of Chemistry, University of Opole, Oleska 48, 45-052 Opole, Poland; radoslaw.balwierz@uni.opole.pl

**Keywords:** caffeic acid phenethyl ester, cytokines, inflammation, in vitro model, polyphenols, propolis

## Abstract

Propolis, owing to its antibacterial and anti-inflammatory properties, acts as a cariostatic agent, capable of preventing the accumulation of dental plaque and inhibiting inflammation. The anti-inflammatory properties of propolis are attributed to caffeic acid phenethyl ester (CAPE), which is present in European propolis. The objective of the conducted study was to assess the anti-inflammatory effects of the Polish ethanolic extract of propolis (EEP) and isolated CAPE on stimulated with LPS and IFN-α, as well as the combination of LPS and IFN-α. The cytotoxicity of the tested compounds was determined using the MTT assay. The concentrations of specific cytokines released by the HGF-1 cell line following treatment with EEP (25–50 µg/mL) or CAPE (25–50 µg/mL) were assessed in the culture supernatant. In the tested concentrations, both CAPE and EEP did not exert cytotoxic effects. Our results demonstrate that CAPE reduces TNF-α and IL-6 in contrast to EEP. Propolis seems effective in stimulating HGF-1 to release IL-6 and IL-8. A statistically significant difference was observed for IL-8 in HGF-1 stimulated by LPS+IFN-α and treated EEP at a concentration of 50 µg/mL (*p* = 0.021201). Moreover, we observed that CAPE demonstrates a stronger interaction with IL-8 compared to EEP, especially when CAPE was administered at a concentration of 50 µg/mL after LPS + IFN-α stimulation (*p* = 0.0005). Analysis of the phenolic profile performed by high-performance liquid chromatography allowed identification and quantification in the EEP sample of six phenolic acids, five flavonoids, and one aromatic ester—CAPE. Propolis and its compound—CAPE—exhibit immunomodulatory properties that influence the inflammatory process. Further studies may contribute to explaining the immunomodulatory action of EEP and CAPE and bring comprehensive conclusions.

## 1. Introduction

Inadequate oral hygiene is closely linked to systemic diseases. Dental plaque significantly influences the progression of various conditions, including cardiovascular diseases, cerebrovascular diseases, and respiratory disorders. Furthermore, the dental biofilm, particularly subgingival plaque, contributes to the development of periodontal disease, which involves intricate interactions of multiple factors. Consequently, periodontal diseases, encompassing gingivitis and periodontitis, have the potential to exacerbate the severity and complications of conditions such as diabetes, insulin resistance, rheumatoid arthritis, obesity, osteoporosis, Alzheimer’s disease, and more recently, COVID-19 [1,2,3,4].

The correlation between periodontal diseases and systemic diseases is underpinned by both direct and indirect mechanisms. The direct mechanism involves the transfer of bacteria into the systemic circulation, leading to bacteriemia in distant organs. The indirect mechanism is associated with inflammatory mediators. Cariogenic bacteria release proteolytic enzymes (such as hyaluronidase, collagenase, and elastase), toxins, and bacterial metabolic products [1]. These factors activate immunocompetent cells, prompting the production and release of inflammatory mediators. Consequently, due to the inflammatory response within periodontal tissues, a range of substances, including metalloproteinases, prostaglandins, eicosanoids, kinins, cytokines, chemokines, and complement activation products, are released into the bloodstream [1,5]. As a result of this process, there is an increase in the levels of pro-inflammatory cytokines in the bloodstream, with interleukins being among the key inflammatory mediators common to both periodontal and systemic disease pathways. Among the primary inflammatory mediators shared by both periodontal and systemic disease pathomechanism are interleukins. The persistent secretion of pro-inflammatory interleukins such as interleukin-1β (IL-1β), interleukin-8 (IL-8), and interleukin-15 (IL-15) contribute to the maintenance of chronic inflammation [5,6,7,8,9,10,11,12].

The interleukins mentioned, along with tumour necrosis factor (TNF-α), constitute the primary proinflammatory cytokines, exerting diverse biological effects on the body [13,14]. An evident correlation exists between dental plaque, the host immune response, and inflammation. Given this intricate relationship, researchers are actively exploring natural substances that can inhibit dental plaque formation and mitigate the release of inflammatory mediators. Propolis, a well-known bee product, has been established to possess confirmed antibacterial and anti-inflammatory properties. Among its bioactive compounds, polyphenols stand out as they demonstrate an antibacterial impact on cariogenic bacteria while also exhibiting anti-inflammatory properties [8,15,16]. Galangin, chrysin, pinobanksin, quercetin, naringenin, apigenin, tt-farnesol, artepillin C, phenolic acids, ursolic acid, and Baccarin collectively contribute to the antibacterial efficacy of propolis. Meanwhile, the anti-inflammatory properties of propolis are attributed to caffeic acid phenethyl ester (CAPE) [15].

Hence, propolis emerges as a promising candidate for averting the buildup of dental plaque while concurrently demonstrating preventive anti-inflammatory effects. Polish propolis is categorized within the European propolis type *Populus*, distinguished by its notable content of a compound endowed with robust biological activity [8]. CAPE effectively modulates various inflammatory pathways, notably by inhibition of the Nuclear Factor kappa B (NF-κB) activity, which plays a pivotal role in the inflammatory process 15.

The aim of this study was to assess the influence of EEP (ethanolic extract of propolis) and isolated CAPE on specific pro-inflammatory cytokines, namely IL-1β, IL-6, IL-8, IL-15, and TNF-α, which are released by HGF-1 (Human Gingival Fibroblasts-1). HGF-1 cells represent a diverse cell population within the periodontium, rendering them an optimal model for examining the effects of propolis and its constituent, CAPE, on the management of oral. We decided to compare the impact of propolis and CAPE on the production of selected proinflammatory cytokines due to CAPE being responsible for the anti-inflammatory effect of propolis [8,15]. The main question of the conducted study was as follows: “What is the immunomodulatory function of propolis and CAPE, as well as their impact on releasing pro-inflammatory cytokines by HGF-1?” The novelty of this study is due to the lack of research describing the secretion of pro-inflammatory cytokines by HGF-1, followed by stimulation with LPS, INF-α, and LPS + INF-α. Our study presented new insights for explaining and understanding the impact of propolis and CAPE on cytokines released by HGF-1 and may increase the dentistry application of propolis. Naeem et al. [17] highlighted the potential innovative application of propolis in the aspect of dentine increased its microhardness over time.

Therefore, the presented study may bring potentially useful knowledge in the field-wide application of propolis in the oral cavity and opens new strategies for future research.

## 2. Results

The focal point of this study was the exploration of the chemical composition, cytotoxic potential, and anti-inflammatory properties inherent in the ethanolic extract of Polish propolis. Furthermore, the study encompassed the assessment of cytotoxicity and anti-inflammatory attributes associated with CAPE.

### 2.1. The Phenolics Content in Ethanol Extract of Propolis

The determination of the phenolic composition of ethanol extract of propolis sample by HPLC provides twelve of twenty-five selected phenolic compounds. The remaining phenolic compounds were present in trace amounts and were not quantified. Among phenolic acids, gallic acid, chlorogenic acid, caffeic acid, *p*-coumaric acid, ferulic acid, and *t*-cinnamic acid were determined. Among flavonoids, luteolin, quercetin, apigenin, chrysin, pinocembrin, and one aromatic ester—CAPE—were determined. The identified phenolic compounds and their content are presented in Table 1. The chemical structures of phenolic compounds detected in sEEP are presented in Table S1. Moreover, the limit of detection (LOD) and limit of quantification (LOQ) of standards are presented in Table S2.

### 2.2. Effect of Ethanolic Extract of Propolis on Viability of HGF-1

**Figure 1:**
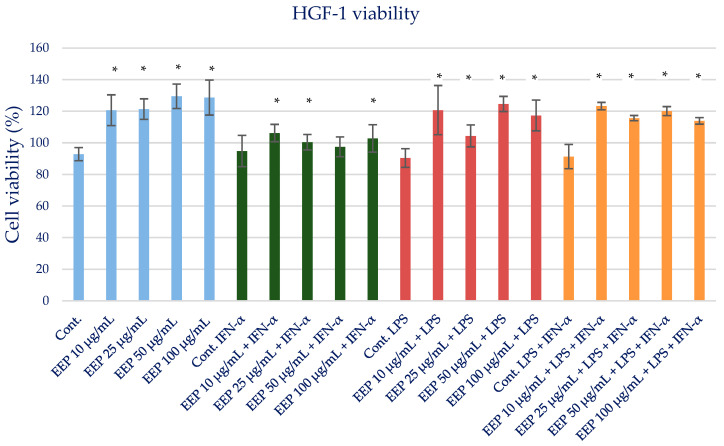
Cell viability measured by MTT (%)—the cytotoxic activity of propolis ethanolic extract. The values represent the mean ± SD of three independent assays; * means *p* < 0.05 (calculated using *t*-test).

The cell viability of the ethanolic extract of Polish propolis was determined using the MTT test. EEP at the tested concentrations in the range of 25–100 µg/mL did not exert a cytotoxic effect. The gingival fibroblast before and after the MTT test is shown in Figures S1 and S2. The results are presented in Table S3 and Figure 1. Therefore, for evaluating anti-inflammatory activity, EEP was used at the proposed two concentrations of 25 and 50 µg/mL and did not exert a cytotoxic effect.

### 2.3. Effect of CAPE on Viability of HGF-1

**Figure 2:**
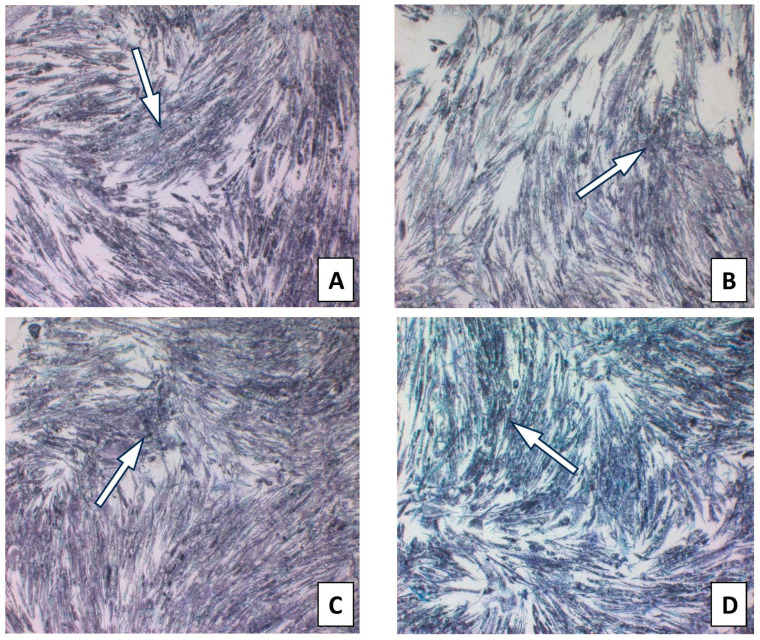
HGF-1 cells with CAPE incubated with MTT for 4 h presenting formazan crystals. (**A**). CAPE 10 µg/mL; (**B**). CAPE 25 µg/mL; (**C**). CAPE 50 µg/mL; (**D**). CAPE 100 µg/mL. (The arrow marks selected formazan crystals).

**Figure 3:**
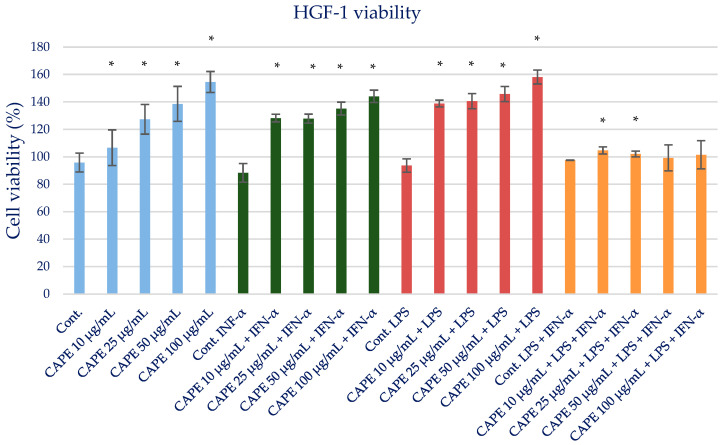
Cell viability measured by MTT (%)—the cytotoxic activity of CAPE. The values represent the mean ± SD of three independent assays; * means *p* < 0.05 (calculated using *t*-test).

The toxicity of isolated CAPE as a major bioactive anti-inflammatory component of propolis was evaluated. The MTT test was used to determine the cell viability of CAPE as an individual propolis component. CAPE at the tested concentrations in the range of 25–100 µg/mL did not exert a cytotoxic effect (Figure 2). The results are presented in Table S4 and Figure 3. Therefore, for anti-inflammatory activity, CAPE was used at the selected concentrations of 25 and 50 µg/mL and did not exert a cytotoxic effect.

### 2.4. Effect of Ethanolic Extract of Propolis on Selected Pro-Inflammatory Cytokines Production in Fibroblast HGF-1 Cells Stimulated by LPS, IFN-α, LPS + IFN-α

**Figure 4:**
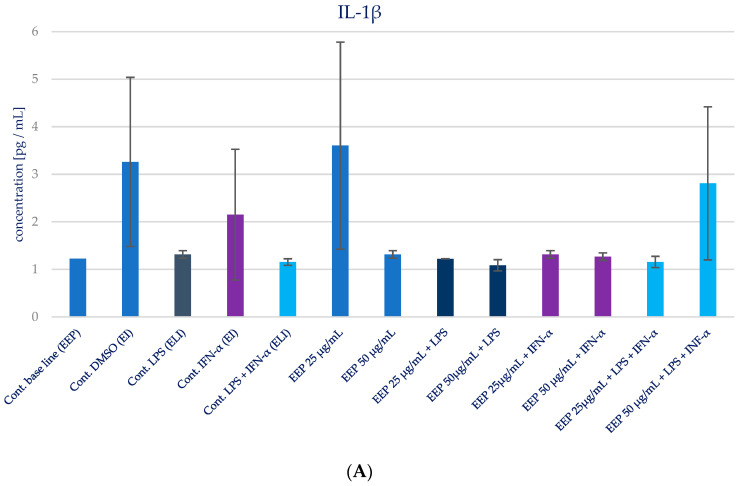

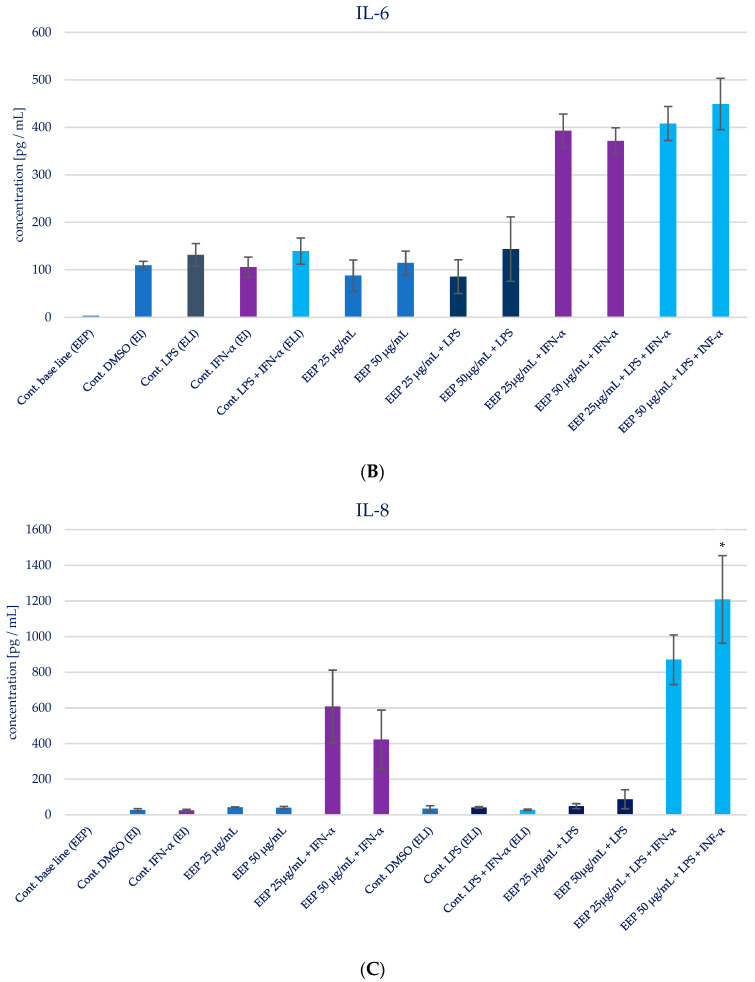

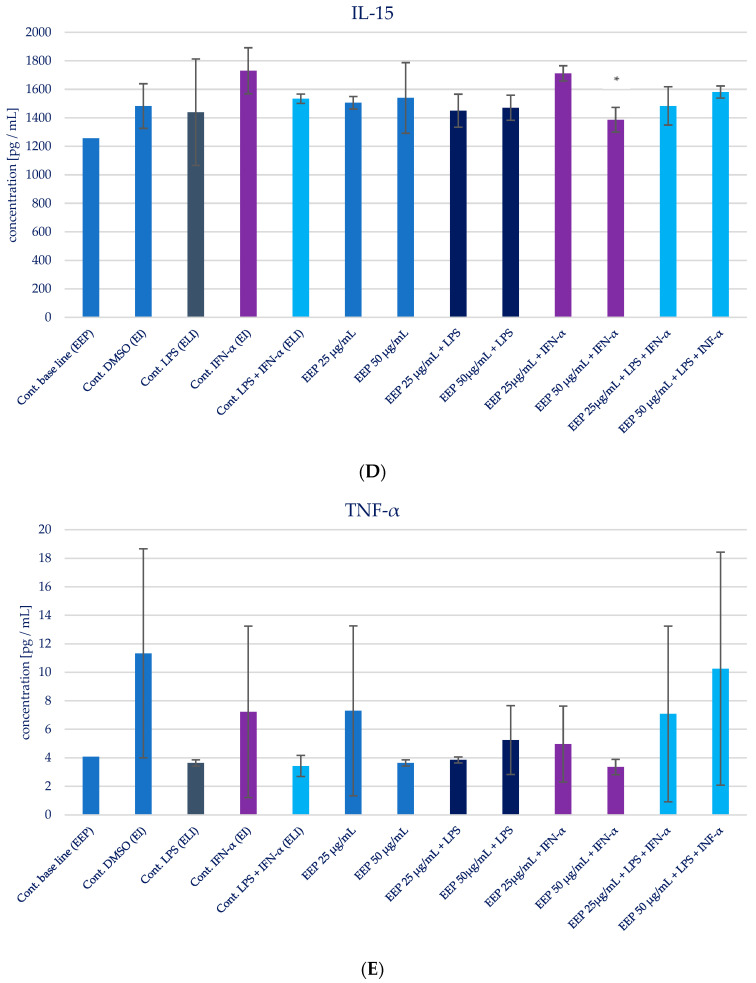
Effect of EEP on selected cytokines production in native and stimulated HGF-1 by LPS, IFN-α, as well as combined LPS and IFN-α. (**A**). IL-1β, (**B**). IL-6, (**C**). IL-8, (**D**). IL-15, (**E**). TNF-α. The presented values mean ± SD of three independent experiments (*n* = 8); * means *p* < 0.05 (calculated using Fisher’s LSD Test).

The impact of Polish propolis ethanolic extract on the generation of specific cytokines (IL-1β, IL-6, IL-8, IL-15, and TNF-α) was examined via experimental conditions involving EEP in LPS, EEP in IFN-α, and EEP in LPS + IFN-α-stimulated HGF-1 cells. The results are depicted in Figure 4 and presented in Table 2. It was anticipated, based on the existing literature, that LPS, IFN-α, and the combined effect of LPS + IFN-α would trigger elevated secretion of the designated pro-inflammatory cytokines in comparison to the cytokine levels observed in the control cell line.

During the evaluation of the effects of EEP at concentrations of 25 and 50 µg/mL on cytokines production in HGF-1 fibroblast cells exposed to LPS, IFN-α, and EEP in the LPS + IFN-α stimulation, certain variations were observed. However, these variations did not achieve statistical significance, particularly with regard to IL-1β. A similar pattern emerged in relation to TNF-α levels, where EEP did not induce significant changes. Notably, EEP, at both concentrations, resulted in an increase in IL-6 and IL-8 secretion, as illustrated in Figure 4B,C. In the context of EEP treatment, in both concentrations, an elevation in IL-6 and IL-8 secretion was noted following IFN-α stimulation and LPS + IFN-α stimulation. Moreover, a statistically significant difference was observed for IL-8 when EEP was administered at a concentration of 50 µg/mL in HGF-1 cells stimulated by LPS + IFN-α (*p* = 0.021201). Regarding the impact of EEP on IL-15, noteworthy shifts in IL-15 levels were identified at a concentration of 50 µg/mL following IFN-α stimulation (*p* = 0.001215), as depicted in Figure 4D. Additionally, IL-15 exhibited the least noticeable differentiation among all the analyzed cytokines.

### 2.5. Effect of CAPE on Selected Pro-Inflammatory Cytokines Production in Fibroblast HGF-1 Cells Stimulated by LPS, IFN-α, and LPS + IFN-α

**Figure 5:**
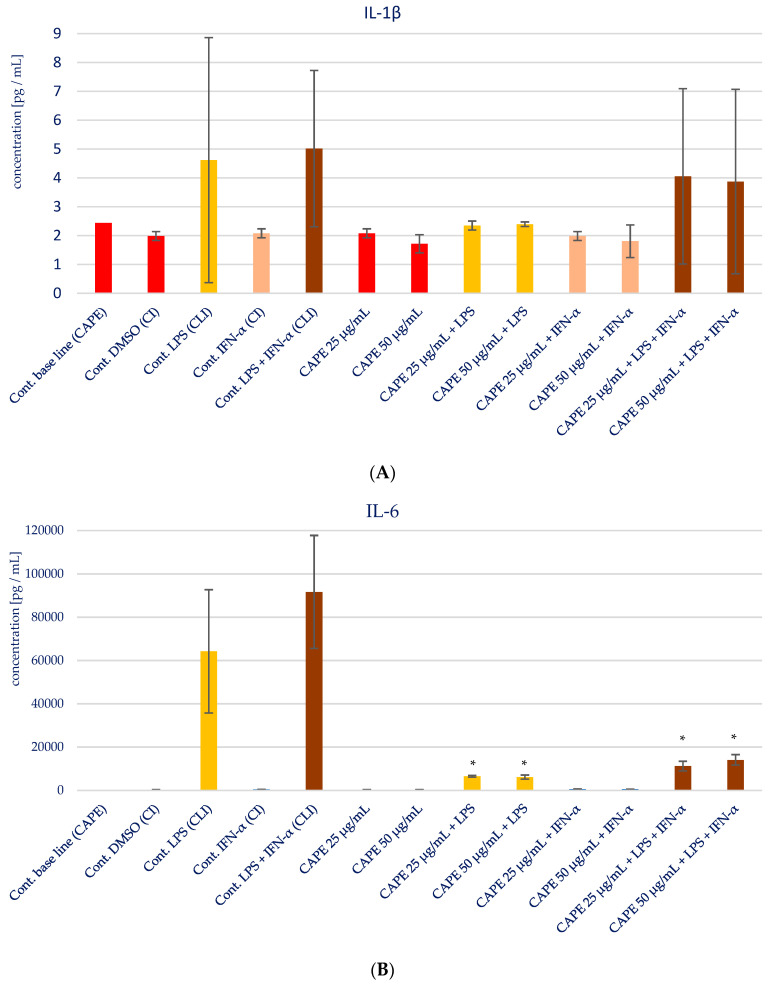

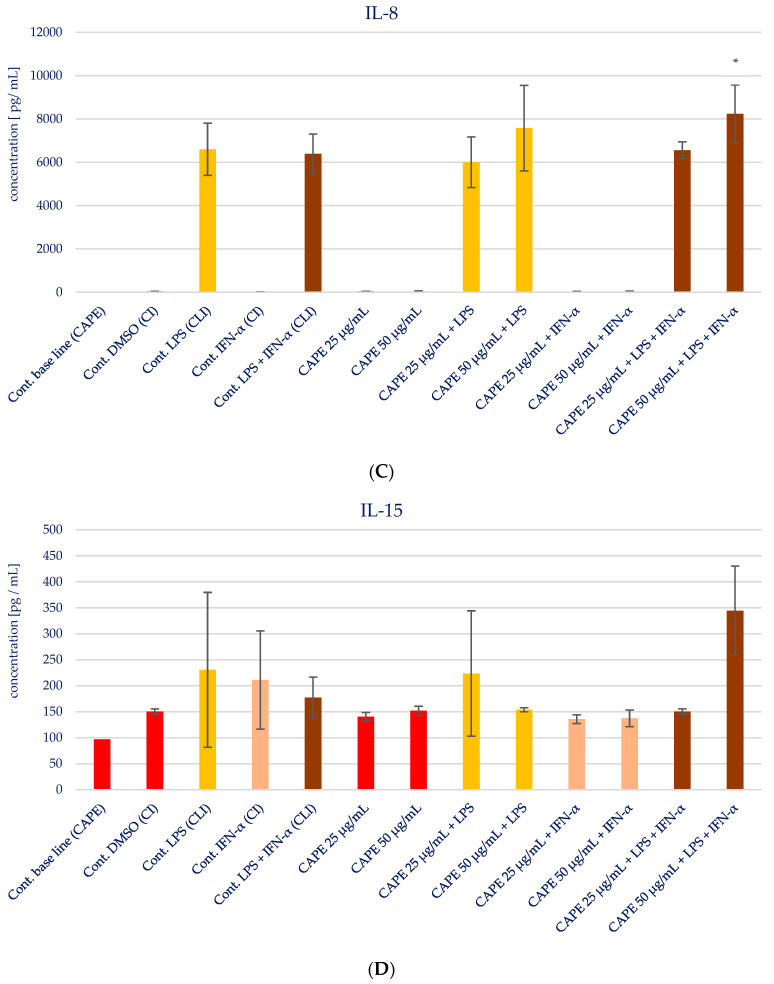

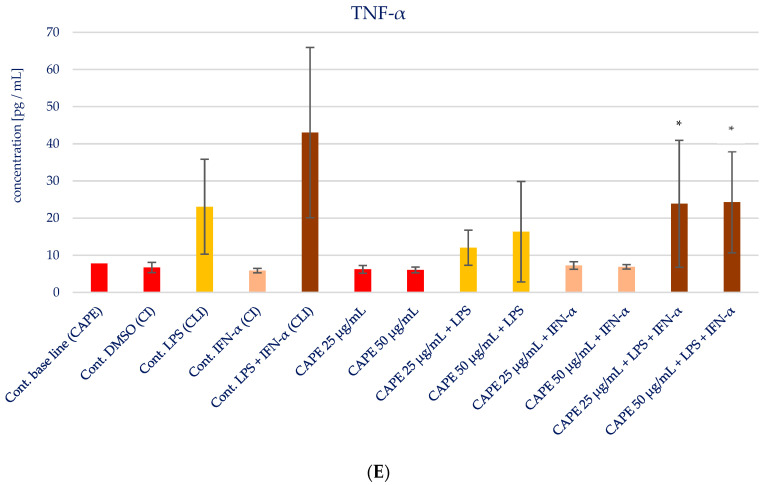
Effect of CAPE on selected cytokines production in native and stimulated HGF-1 by LPS and IFN-α, as well as combined LPS and IFN-α; * means *p* < 0.05 (calculated using Fisher’s LSD Test).

The impact of CAPE on the production of specific pro-inflammatory cytokines (IL-1β, IL-6, IL-8, IL-15, and TNF-α) in various stimulatory conditions, including LPS, IFN-α, and the combination of LPS and IFN-α, is illustrated in Figure 5 and presented in Table 3. Upon analyzing the influence of CAPE on cytokines production in HGF-1 fibroblast cells stimulated with LPS, IFN-α, and LPS + IFN-α, no noticeable changes were observed for IL-1β, similar to the results seen with EEP. However, statistically significant variations were noted in the concentration of IL-6 following CAPE treatment at both concentrations, post-stimulation with LPS and LPS + IFN-α in HGF-1 cells (*p* = 0.000). Similar to the outcomes observed with EEP, the administration of CAPE led to an increase in the concentration of IL-8. Notably, a statistically significant increase was seen when CAPE was administered at a concentration of 50 µg/mL after LPS + IFN-α stimulation (*p* = 0.0005). IL-15 exhibited the least distinct changes, as no statistically significant differences were observed, in contrast to EEP, where it was observed. Nonetheless, CAPE induced a reduction in the concentration of TNF-α, particularly evident following HGF-1 cell stimulation with LPS + IFN-α (*p* = 0.0045 and *p* = 0.0054), as illustrated in Figure 5E.

### 2.6. Statistical Analysis Comparing the Impact of EEP and CAPE on Cytokines Production in Fibroblasts HGF-1 Cells Stimulated by LPS, IFN-α, and LPS + IFN-α

Based on our findings, it is found that CAPE exhibits a clear inhibitory effect on TNF-α and IL-6, particularly in sequences where HGF-1 cells are stimulated with LPS or a combination of LPS and IFN-α. Conversely, we observed that EEP demonstrates a stimulatory effect on the production of IL-6 and IL-8. In the case of CAPE, its stimulatory action is primarily limited to IL-8. In light of these observations, we proceeded to subject all data to further investigation using more advanced statistical methods.

**Figure 6:**
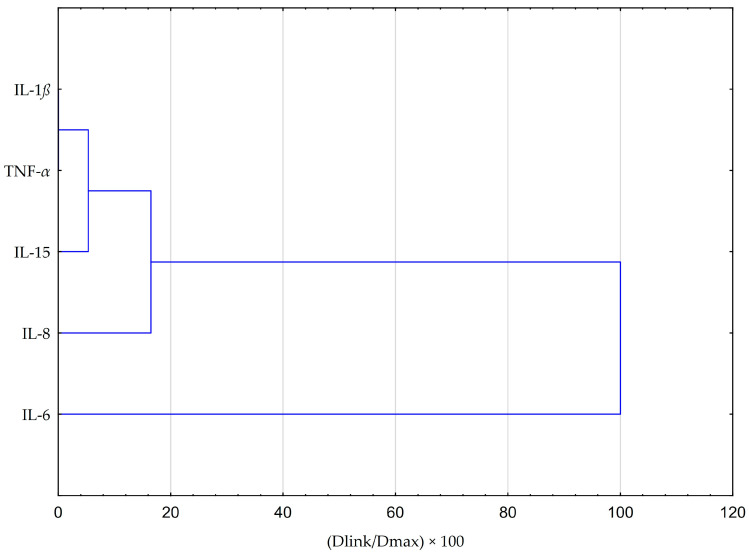
Dendrogram obtained via the HCA analysis of all obtained data based on the average content of the effect of EEP and CAPE at different concentrations on pro-inflammatory cytokines.

**Figure 7:**
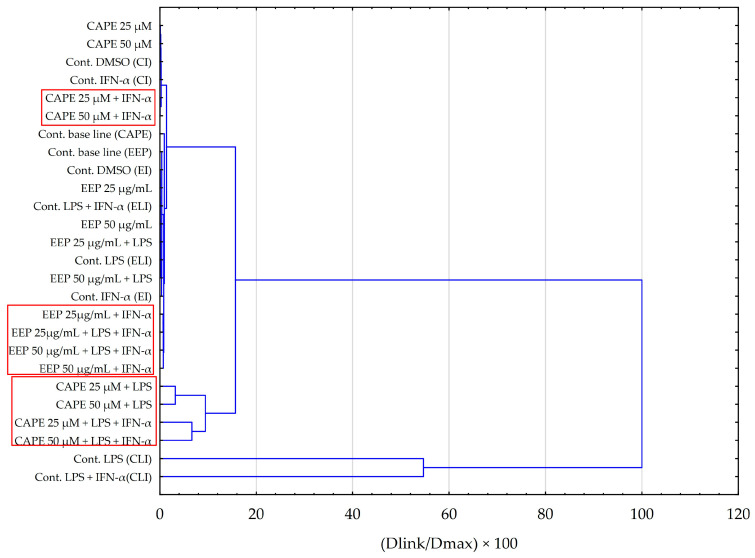
Dendrogram obtained via the HCA analysis of all obtained data based on the influence on pro-inflammatory cytokines by the average content of the EEP and the CAPE at different concentrations.

**Figure 8:**
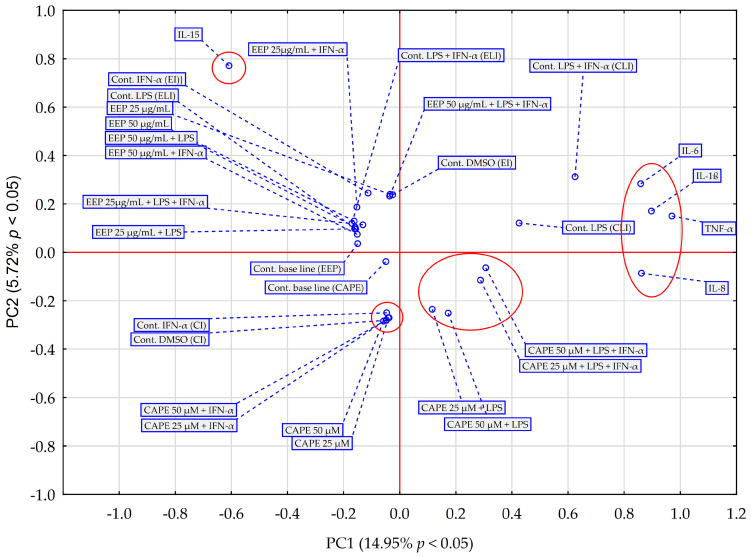
PCA score plot of all obtained data based on the average content of the effect of EEP and CAPE at different concentrations on pro-inflammatory cytokines. PC—principal component.

Principal component analysis (PCA) and hierarchical clustering analysis (HCA) using Euclidean distances were employed to analyze the impact of EEP and CAPE on the concentrations of pro-inflammatory cytokines evaluated in HGF-1 cells stimulated by LPS, IFN-α, and LPS + IFN-α. The results obtained from the HCA analysis are presented in Figure 6 and Figure 7, while the results from the PCA analysis are shown in Figure 8.

The HCA was based on the Euclidean distance defined between sets. The obtained dendrogram of cytokines demonstrated that all data could be clustered into three main groups (Figure 6). The first cluster group indicates similarities in changes in IL-1β and TNF-α concentrations. The depicted effects are illustrated in Figure 6 and Figure 7, revealing a resemblance in the impact of both EEP and CAPE on these cytokines. Nevertheless, the statistical significance of CAPE’s effect obtained for TNF-α surpasses the effect of EEP (refer to the LSD test results presented in Table 2 and Table 3). In the second cluster, IL-15 and IL-8 demonstrate comparable behaviour concerning the tested samples. Concerning IL-15, a statistically significant decrease in concentration was observed when fibroblasts were stimulated with IFN-α and treated with EEP at a concentration of 50 µg/mL. A different effect was noted for CAPE, although the outcome did not reach statistical significance. Moreover, EEP and CAPE at a concentration of 50 µg/mL for LPS + IFN-α-stimulated fibroblasts caused similar changes in IL-8 concentration (concentration increased with EEP and CAPE at 50 µg/mL). Among the cytokines, IL-6 stands out as the least similar to the other tested cytokines in terms of treatment effects observed for CAPE and EEP. Notably, it demonstrates a notable nine-fold decrease in concentration following CAPE application across all tested concentrations in the context of fibroblasts stimulated by LPS and IFN-α. Conversely, when considering EEP treatment, distinct results emerge, with a three-fold increase observed; however, statistical significance was not established in Fisher’s LSD test.

The hierarchical clustering analysis obtained for tested samples of EEP and CAPE (Figure 7) indicates that similar outcomes were achieved for the stimulation of HGF-1 by LPS and LPS + IFN-α in the case of CAPE. This observation aligns with the findings from the ANOVA test conducted for IL-6. Additionally, it is noteworthy that fibroblasts treated with CAPE and solely stimulated with IFN-α do not exhibit a shared cluster. In the case of EEP, we can observe the formation of distinct clusters between fibroblasts with EEP when stimulated by LPS in combination with IFN-α, as well as when stimulated solely by IFN-α. Nevertheless, the observed effects are contingent upon the concentration of EEP. The analysis appears to corroborate previous data that more favourable outcomes are achieved when utilizing a concentration of 50 µg/mL.

The PCA score plot was generated considering the average impact of various concentrations of EEP and CAPE on pro-inflammatory cytokines (Figure 8). The first two principal components (PCs) explained 20.67% of the variance in the data (14.95% for PC1 and 5.72% for PC2). The principal component analysis (PCA) reveals data indicating that, across all tested stimulation variants, IL-15 exhibits distinct behaviour compared to EEP and CAPE concerning the other pro-inflammatory cytokines under discussion in this study. Notably, IL-15 is a cytokine that frequently maintains its concentration regardless of the method of treatment used; in most cases, changes are statistically insignificant (based on post hoc analysis of the ANOVA test), with an exception observed in fibroblasts stimulated by EEP at concentration 50 µg/mL together with IFN-α, where statistical significance was demonstrated in Fisher’s LSD test. Moreover, IL-15 is a cytokine that EEP affects dissimilarly compared to CAPE. Regarding other pro-inflammatory cytokines, CAPE demonstrates a stronger interaction with IL-8 compared to EEP. This disparity is particularly evident in the concentration changes in IL-8 following in fibroblast stimulation with LPS and IFN-α. In contrast, IL-6 and TNF-α exhibit differing behaviour in relation to samples of EEP and CAPE, a distinction that becomes pronounced when fibroblasts are subjected to dual stimulation by LPS and IFN-α. The analysis also yields insights into the fact that the behaviour of four interleukins (excluding IL-15) towards CAPE remains consistent regardless of using concentration. However, it is worth noting that the response to LPS and IFN-α stimulation in comparison to the control sample response is different from the result obtained for fibroblast stimulation only by LPS; statistical significance was attained in the LSD test for fibroblasts stimulated by LPS and IFN-α. This observation appears to reinforce the hypothesis that, concerning EEP, concentration plays a crucial role in achieving the desired effect regarding the analyzed pro-inflammatory cytokines.

## 3. Discussion

Periodontitis is an inflammatory disease associated with inflammatory mediators. During periodontitis, bacteria release chemicals which activate the innate immune system to release proinflammatory cytokines, contributing to more progression of periodontitis. Cytokines are a group of low molecular weight proteins which play the role of signalling molecules. They are released from macrophages, leukocytes, fibroblasts, endothelial cells, keratinocytes, and other cell types. Taking into consideration the impact of cytokines on inflammation and the course of acute phase reaction, they are divided into three groups: pro-inflammatory cytokines, cytokines from a family of interleukin-6, and anti-inflammatory cytokines [1,10,12].

Pro-inflammatory cytokines contribute to the development of inflammation, which is a multi-stage process involving an acute phase and a chronic phase. The acute phase lasts for several dozen seconds to almost 12 h after the stimulus and then goes into the chronic phase [18]. Adequate termination of the acute phase reaction is essential to avoid chronic inflammation. The immunomodulatory properties of natural products may influence the concentration of pro-inflammatory cytokines and may have a potential impact on resolve of acute inflammation and prevention of chronic inflammation [10,19,20].

Therefore, the anti-inflammatory effect of EEP and isolated CAPE was investigated. We decided to choose CAPE because the European propolis to which propolis from Poland belongs is rich in this component. The chemical analysis of propolis demonstrated that the Polish ethanol extract of propolis contained 320.344 µg CAPE per 1 g of propolis. CAPE exhibits wide biological activity, including the anti-inflammatory action [15]. HGF-1 was chosen as an ideal model for investigating the impact of propolis and an individual component of propolis—CAPE—on oral health management because they are present in the periodontium. The native HGF-1 stimulated by IFN-α, LPS, and combined IFN-α and LPS were incubated with and without EEP or CAPE for 24 h. Based on the literature, it is expected that LPS, IFN-α, and both LPS + IFN-α stimulate the secretion of selected pro-inflammatory cytokines compared to cytokine levels observed in the control line [19]. Our results showed that endotoxin and interferon stimulate the release of pro-inflammatory cytokines by HGF-1 (Figure 4 and Figure 5). It is worth highlighting that the response to LPS and IFN-α stimulation in comparison to the control sample response is different from the result obtained for fibroblast stimulation only by LPS; statistical significance was attained in the LSD test for fibroblasts stimulated by LPS and IFN-α. This observation appears to reinforce the hypothesis that concerning the used agent’s influence effect on the analyzed pro-inflammatory cytokines [20].

Selected cytokines detected in the supernatant of the HGF-1, such as IL-1β, IL-6, IL-8, IL-15, or TNF-α, are important pro-inflammatory mediators in the host-defence response and immune regulation. IL-1β belongs to the family of IL-1 cytokines [10]. It is produced as pro-IL-1β, a precursor protein form. The proteolytic cleavage of pro-IL-1β occurs with caspase-1 (also termed IL-1β-converting enzyme, (ICE)) in inflammasome [11]. Mature IL-1β is secreted mainly by monocytes and macrophages but also by other cells, like fibroblasts. The immune cells produce this cytokine in response to DAMPs (damage-associated molecular patterns), e.g., extracellular ATP or microbial components PAMPs (pathogen-associated molecular patterns), e.g., LPS (lipopolysaccharide) [12]. IL-β plays a key role in the acute phase response of inflammation via the induction of IL-6 and activation of C-reactive protein and complement components; therefore, IL-6 was chosen as an important molecule to determine the action of natural products. IL-6 exhibits pleiotropic action. It is responsible for the regulation of the acute-phase response and chronic inflammation [21]. IL-6 is recognized as a pro-inflammatory cytokine, although it possesses also anti-inflammatory activities [22]. On one hand, an increase in IL-6 concentration as a consequence of the immune system’s response to infectious factors leads to the acute phase of inflammation, while persistent production of mentioned interleukin leads to chronic inflammation. The pro-inflammatory action of IL-6 is mediated by trans-signalling. On the other hand, as underlined by Scheller et al. [23], IL-6 classic signalling, which is based on the stimulation of target cells via a membrane-bound interleukin-6 receptor, mediates the activation of anti-inflammatory pathways on target cells. Taking into consideration the impact of EEP and CAPE on IL-6, we noticed the different impacts of EEP and CAPE. EEP caused an increase in IL-6 concentration, while CAPE caused a decrease in IL-6 concentration. Zamarrenho et al. [20] indicated that propolis reduces the concentration of IL-6; however, differences among studies may be connected to different protocols of the conducted experiment, including timing of LPS or IFN-α stimulation. As was mentioned by Zamarrenho et al. [20], CAPE in low concentrations caused an increase in IL-6, but at ranges of 50 to 100 µg/mL caused a decrease in IL-6. Our study confirmed that CAPE at concentrations 25 and 50 µg/mL exhibits an inhibitory effect on IL-6, particularly in sequences where HGF-1 cells are stimulated with LPS or a combination of LPS and IFN-α.

In our study, IL-8 was assayed because it is an important protein related to inflammation. The enhanced IL-8 expression may be attributed to chronic periodontitis [23,24].

IL-8 is necessary for the migration and action of neutrophils, which take part in acute and chronic inflammation [25].

We observed that CAPE demonstrates a stronger interaction with IL-8 compared to EEP. This disparity is particularly evident in the concentration changes in IL-8 following fibroblast stimulation with LPS and IFN-α that after stimulation of HGF-1 by LPS + IFN-α, EEP at a concentration of 50 µg/mL caused the highest concentration of IL-8. A similar situation was observed in the case of CAPE in the same concentration. Despite the fact that EEP and CAPE increased the concentration of IL-8, this situation may be correlated with the mobilization of the immune system to defence. The results obtained in the present study indicate the immunomodulatory properties of propolis and CAPE. In the presented experiment, a limitation was identified, such as the evaluation of cytokines levels in a short time after stimulation of HGF-1. The experiment was conducted after 24 h, so it seems necessary to continue our study for a longer period of time. However, prolonged elevated expression of IL-8 may be associated with chronic inflammation.

In our investigation, we assayed a concentration of IL-15. Il-15 belongs to pleiotropic cytokines, which play a pivotal role in the development of the inflammatory process and influence the response of the immune system. It takes part in viral and bacterial infections. Among patients with hepatitis C virus treatment with IFN-α, increased concentration of IL-15 was found among patients with chronic hepatitis as well as patients with liver cirrhosis. The highest concentration of IL-15 appeared among patients with hepatocellular carcinoma [26].

Taking into consideration this fact, we analyzed the impact of EEP and CAPE on the concentration of IL-15 after stimulation of IFN-α. However, we noticed that IL-15 is a cytokine that EEP affects dissimilarly compared to CAPE. A statistically significant decrease in the concentration of IL-15 was observed when fibroblasts were stimulated with IFN-α and treated with EEP at a concentration of 50 µg/mL. A different effect was noted for CAPE, although the outcome did not reach statistical significance.

The last assayed cytokine, which is released after infection, as well as exposure to bacterial-derived LPS, was TNF-α. It is enumerated as the most abundant early signalling molecule during inflammation. Scientists emphasize that the excessive production of TNF-α is correlated with chronic inflammation [27,28]. Increased concentrations of TNF-α in patients suffering from periodontitis is closely associated with the destruction of connective tissue and immune response [28,29]. Singh P. et al. [30] observed an increased concentration of TNF-α in saliva among patients with chronic periodontitis. Moreover, diabetes and smoking cause the highest concentration of TNF-α in chronic periodontitis. In our experiment, CAPE reduced the concentration of TNF-α, particularly evident following HGF-1 cell stimulated 4 h with LPS + IFN-α. In the case of EEP, significant changes in the concentration of TNF-α were not observed. A similar situation was noticed in the macrophages model for Brazilian green propolis extract [20].

The anti-inflammatory and immunomodulatory effects of Polish propolis should be investigated both in vitro and in vivo. Polish propolis is rich in CAPE, which is responsible for anti-inflammatory action [8]. CAPE reduced TNF-α and IL-6 in contrast to EEP and CAPE at a concentration of 50 µg/mL after LPS + IFN-α stimulation exhibited stronger interaction with IL-8 compared to EEP. The immunomodulatory potential of propolis and CAPE is varied but may increase the immune response.

To summarize our study, it is worth highlighting that understanding the effect of cytokines released by gingival fibroblasts may allow us to propose a new strategy of oral disease therapy. The prevention of caries or periodontal disease is achieved by not allowing cariogenic microorganisms or periodontopathogens to colonize the enamel surface. This is possible by using toothpastes or lozenges with propolis, the active immunomodulatory ingredient. Strengthening the host defence by propolis within the oral cavity against colonization by Gram-negative bacteria or viral infections can help maintain plaque homeostasis. Ending the acute inflammatory phase by effectively mobilizing the immune system is essential to avoid chronic inflammation. CAPE as a single ingredient does not act—as it is composed of numerous active components—as propolis does. Propolis is a mixture of different phenolic compounds. We observed changes in the concentration of cytokines after 24 h, but further studies are needed to explain clearly the impact of propolis and CAPE in late time, e.g., after 72 h.

## 4. Materials and Methods

### 4.1. Materials

The Polish propolis sample was delivered from an apiary in Kamianna, Poland. CAPE of synthetic origin was delivered from Sigma Aldrich (Munich, Germany). Ethanol 96% was purchased from POCH (Gliwice, Poland). DMSO was purchased from Sigma Chemical Company (St. Louis, MO, USA). LPS was purchased from Fluka Chemie GmbH (Buchs, Switzerland); thus, IFN-α was purchased from Roche Company, Poland. The human gingival fibroblast cell was obtained from the American Type Culture Collection (ATCC, Manassas, VA, USA). Dulbecco’s Modified Eagle’s Medium (DMEM), Fetal Bovine Serum (FBS), and Trypsin-EDTA were purchased from Sigma Aldrich (Darmstadt, Germany). All standards used in the RP-HPLC-PDA analysis were purchased from Sigma Aldrich (Munich, Germany). Gallic acid, protocatechuic acid, chlorogenic acid, *p*-OH benzoic acid, caffeic acid, syringic acid, *m*-OH benzoic acid, ellagic acid, *p*-coumaric acid, and ferulic acid were dissolved in 50–50% methanol-pure water. Other standards, such as epicatechin, rutin, myricetin, resveratrol, daidzein, luteolin, quercetin, *t*-cinnamic acid, apigenin, hesperetin, rhamnetin, chrysin, pinocembrin, CAPE, and curcumin were dissolved in 100% methanol. Methanol and acetonitrile, as well as acetic acid, were obtained from MERCK (Darmstadt, Germany).

### 4.2. Ethanol Extract of Propolis Formulation

The solid ethanol extract of propolis (sEEP) was prepared based on the raw propolis, which was obtained from the “Barć” apiary in Kamianna, Poland. The 100 g of raw propolis was mixed with 1 L of 70% ethanol and stirred for 24 h. Next, the extraction mixture was filtered under a vacuum through the paper filter, the filtrate was collected, and the sediment was mixed again with 0.5 L of 70% ethanol and stirred for 24 h. After this, the filtration proceeded under the conditions above. The filtrate was added to the previous filtrate, and the sediment was finally extracted with 0.1 L of 70% ethanol (and filtered in previous conditions). The third extract was added to previously obtained extracts. Joined extracts were solid in the vacuum in two steps at 40 °C. At first, the extract was concentrated to the consistency of thick syrup on a rotary vacuum evaporator (IKA-Werke RV 05 Basic). Then, the concentrated extract was evaporated to dryness in a vacuum oven (Infitek DOV-25H). Finally, the EEP was diluted in DMSO at concentrations of 10, 25, 50 and 100 µg/mL.

### 4.3. RP-HPLC-PDA Analysis

The phenolic composition of each sample was analyzed using a modified method as described by Can et al. [31]. High-performance liquid chromatography (HPLC) analyses were performed using a Shimadzu Liquid Corporation LC 20AT HPLC system, which was equipped with a photodiode array (PDA) detector. A C18 column with dimensions of 250 mm × 4.6 mm and a particle size of 5 μm, provided by GL Sciences, was utilized in the analysis [32]. The elution process was conducted using a gradient program: mobile phase A, which consisted of a 70% acetonitrile–ultra-pure water solution, and mobile phase B, which was a 2% acetic acid in water solution. The flow rate during the analysis was set at 1 mL/min, with an injection volume of 20 μL. The column temperature was maintained at 30 °C throughout the analysis. The detection range spanned from 250 nm to 360 nm, with wavelengths specifically monitored at 250 nm, 280 nm, 320 nm, and 360 nm. The confirmation of phenolic compounds in the samples and the determination of their concentrations were conducted by comparing the retention times with those of actual standards and by analyzing the ultraviolet absorption spectrum data. Before the analysis, all prepared samples underwent filtration using 0.45 µm membranes before being injected into the device [32].

### 4.4. HGF-1 Collection

The cultivation of HGF-1 was performed in a flask of 25 mL under sterile conditions. The commercial medium growth Dulbecco’s Modified Eagle’s Medium (DMEM) with L-glutamine, which was modified by ATTC to contain 4.5 g/L glucose, 1.5 g/L sodium bicarbonate, supplemented with 10% fetal bovine serum (FBS) was used. In order to ensure proper growth, 100 U/mL penicillin and 100 µg/mL streptomycin were added to the growth medium. The medium was formulated for use with 5% CO_2_ in the incubator. Cells were cultured at 37 °C. They were detached with trypsin-EDTA at a concentration of 0.05% for 3 min. In order to determine the number of cells, we used Bürker’s counting chamber using Formula (1).

(1) $$\mathrm{Number}\;\mathrm{of}\;\mathrm{cells}\;\mathrm{in}\;1\;\mathrm{mL}=\frac{4\;\mathrm{squares}\;\mathrm{counted}}{2}\times 100\times 1000$$

The number of cells was diluted to 100,000 cells/mL. An amount of 200 µL of the cultured cells was seeded in 96-well plates in the presence of LPS and IFN-α with or without EEP for 24 h. In the experiment, HGF-1 was exposed to EEP at concentrations of 10, 25, 50, and 100 µg/mL and CAPE at concentrations of 10, 25, 50, and 100 µg/mL.

### 4.5. Cell Viability Assay

The cell viability was determined by the MTT test. This method is based on the reduction of 3-(4,5-dimethyl-2-thiazyl)-2,5-diphenyl-2H-tetrazolium bromide. In this reaction, tetrazolium salt MTT is transferred to a blue formazan crystal by viable cells. EEP at the final concentrations of 10, 25, 50, and 100 µg/mL with or without LPS, IFN-α, and LPS combined with IFN-α was added to 96 wells, such that 1 well contained 200 µL. After 24 h, the medium was removed, and 180 µL medium and 20 µL MTT solution (5 mg/mL PBS) were added to each well. The time of incubation was 4 h. Then, the supernatant was removed, and DMSO was added to each well in order to dissolve the obtained formazan crystals. The controls contain native cells and medium alone. The estimation was made spectrophotometrically at 550 nm wavelength.

The results expressed as absorbance were calculated according to the following formula [33]:

% cell viability = sample absorbance × 100/absorbance of the control.

### 4.6. Multiplex Bead-Based Cytokine Assay

Five selected pro-inflammatory cytokines released from the HGF-1 cell line treated with EEP (25–50 µg/mL) were assayed in the culture supernatant. Multiplex assay was applied for cytokine detection. Bio-Plex Human cytokine Panel (BIO-RAD) was used using Bio-Plex (version 5.0) 200 System based on xMAP technology (BIO-RAD Laboratories Inc., Hercules, CA, USA). The native HGF-1 (1 × 10^6^/mL) stimulated by IFN-α, LPS, and combined IFN-α and LPS were incubated with and without EEP at range concentrations of 25–50 µg/mL for 24 h. Standard dilution series and blank were prepared using kit-supplied references of selected cytokines. The assay was led in a single well using 50 µL of EEP. The supernatant obtained from the HGF-1 culture line was incubated with antibody-conjugated magnetic beads for 30 min and washed with buffer. After this step, biotinylated detection antibodies and streptavidin–phycoerythrin conjugates were added to each well. The time for incubation was 30 min. Next, unbounded streptavidin was removed by washing with buffer. Finally, selected cytokines bounded with beads were determined in the Bio-Plex Array Reader (Bio-Plex 200 System). The fluorescence was measured using Bio-Plex Manager software, Version 5.0 (BIO-RAD) [34].

### 4.7. Statistical Analysis

All the analyses were carried out in triplicate, and the results were expressed as means. The statistical analyses were performed using STATISTICA 13.1 software (StatSoft Inc., Tulsa, OK, USA). The obtained data were analyzed using hierarchical cluster analysis (full linkage using Euclidean distance) and principal component analysis (PCA). The PCA model was estimated using the NIPALS iterative algorithm. The criterion of convergence was set at the level of 0.00001, and the maximum number of iterations was set at 50. The number of components was determined by determining the maximum predictive capability using the method of multiple cross-validations, and the maximum number of components was set at the level. The obtained optimal PCA model was then reduced to 2 components. The conducted PCA, the results of which are presented on the chart of PC 1 vs. PC 2 loads, allowed us to select variables with the most significant influence on the variability of the analyzed database of results and to select the most significant correlations between them. These two classification techniques (PCA and HCA) were used to discover natural groupings in the data and examine differences between the analyzed influences of CAPE and EEP into HGF-1 fibroblasts. A one-way ANOVA was used to compare the effect of CAPE and EEP at different concentrations on the production of pro-inflammatory cytokines stimulated by LPS, IFN-α, and combined LPS and IFN-α. A *t*-test was used to evaluate the MTT test. Statistical significance was considered when *p* < 0.05.

## 5. Conclusions

Propolis and its compound—CAPE—exhibit immunomodulatory properties that influence the inflammatory process. Propolis did not induce significant changes in the concentration of IL-1β and TNF-α. Based on the conducted study, CAPE did not induce significant changes in the concentration of IL-1β; however, it reduced TNF-α and IL-6 in contrast to EEP. Propolis seems effective in stimulating HGF-1 to release IL-6 and IL-8. The synthesis of pro-inflammatory cytokines in the gingival fibroblast stimulated with endotoxin-lipopolysaccharide and protein interferon-α and treated with propolis or CAPE influence the course of acute inflammation. Additionally, CAPE demonstrates a stronger interaction with IL-8 compared to EEP, especially when CAPE was administered at a concentration of 50 µg/mL after LPS + IFN-α stimulation. Therefore, it enhances the immune response and may contribute to ongoing inflammation. Substantial termination of the acute inflammatory phase via efficient mobilization of the immune system is essential to avoid chronic inflammation. Therefore, it would be unreasonable to continue the research to confirm the effect of propolis and CAPE on the concentration of pro-inflammatory cytokines after a longer period of time, e.g., after 72 h. The obtained results demonstrate that in the case of EEP, the concentration is significant for the effect obtained in terms of the analyzed pro-inflammatory cytokines. At higher concentrations of propolis, a stronger increase in the release of pro-inflammatory cytokines was observed. Further studies may contribute to explaining the immunomodulatory action of EEP and CAPE and bring comprehensive conclusions.

## Figures and Tables

**Table 1:** Phenolic compounds detected in solid ethanolic extract of propolis (sEEP) ((µg phenolic/g sEEP), ±SD, *n* = 3) obtained by HPLC analysis. Statistical significance was calculated using *t*-test (*p* < 0.05).

No	Phenolic Compounds	Content [µg Phenolic/g EEP]	*p*
1.	gallic acid	12.789 ± 0.170	0.000
2.	protocatechuic acid	-	-
3.	chlorogenic acid	11.022 ± 0.02	0.000
4.	*p*-OH Benzoic acid	-	-
5.	epicatechin	-	-
6.	caffeic acid	320.344 ± 1.14	0.001
7.	syringic acid	-	-
8.	*m*-OH benzoic acid	-	-
9.	routine	-	-
10.	ellagic acid	-	-
11.	*p*-coumaric acid	3002.604 ± 32.06	0.009
12.	ferulic acid	318.816 ± 18.01	0.002
13.	myricetin	-	-
14.	resveratrol	-	-
15.	daidzein	-	-
16.	luteolin	8.799 ± 0.08	0.000
17.	quercetin	90.700 ± 2.1	0.000
18.	*t*-cinnamic acid	59.210 ± 1.06	0.002
19.	apigenin	328.005 ± 12.3	0.003
20.	hesperitin	-	-
21.	rhamnetin	-	-
22.	chrysin	1998.700 ± 56.4	0.005
23.	pinocembrin	7.899 ± 0.7	0.000
24.	CAPE	989.699 ± 1.82	0.000
25.	curcumin	-	-

**Table 2:** The effect of propolis ethanolic extract on the production of selected cytokines, i.e. IL-1β, IL-6, IL-8, IL-15, TNF-α and EEP in LPS, EEP in IFN-α, as well as EEP in LPS + IFN-α stimulated HGF-1 cells (*n* = 3). Statistical significance was analyzed using Fisher’s LSD test. Results marked in bold are statistically significant in Fisher’s LSD test. Multivariate tests of significance (F = 12.91, *p* < 0.05).

Sample	IL-1β			IL-6			IL-8			IL-15			TNF-α		
	AVG	SD	*p*	AVG	SD	*p*	AVG	SD	*p*	AVG	SD	*p*	AVG	SD	*p*
Control baseline (EEP)	1.224			3.410			1.360			1256.310			4.070		
Control DMSO (EI)	3.261	1.778		109.283	8.940		27.600	6.946		1482.840	156.863		11.330	7.335	
Control LPS (ELI)	1.315	0.079		131.800	23.512		41.693	4.151		1439.930	373.413		3.642	0.214	
Control IFN-α (EI)	2.153	1.374		105.797	21.038		24.810	5.595		1730.793	161.382		7.222	6.018	
Control LPS + IFN-α (ELI)	1.156	0.068		139.400	27.655		27.477	4.218		1534.390	32.684		3.427	0.742	
EEP 25 µg/mL	3.603	2.178		87.747	33.217		42.423	1.686		1505.647	44.126		7.294	5.959	
EEP 50 µg/mL	1.315	0.079		114.503	25.121		40.233	6.905		1539.677	248.184		3.642	0.214	
EEP 25 µg/mL + LPS	1.224	0.000	0.9442	85.727	35.643	0.9941	48.643	13.891	0.9889	1450.367	116.122	0.9178	3.856	0.214	0.9736
EEP 50 µg/mL + LPS	1.088	0.118	0.8611	143.770	67.882	0.9985	87.427	53.326	0.9271	1470.890	87.826	0.7596	5.251	2.416	0.8040
EEP 25 µg/mL + IFN-α	1.315	0.079	0.5182	392.797	35.264	0.9633	608.307	204.011	0.2460	1711.937	53.431	0.8521	4.965	2.663	0.7277
EEP 50 µg/mL + IFN-α	1.269	0.079	0.4960	371.620	27.279	0.9660	422.833	164.744	0.4271	1386.310	86.914	**0.0012**	3.356	0.539	0.5514
EEP 25 µg/mL + LPS + IFN-α	1.156	0.118	1.0000	408.203	35.695	0.9656	870.680	139.463	0.0959	1483.853	134.711	0.6177	7.080	6.164	0.5736
EEP 50 µg/mL + LPS + IFN-α	2.811	1.610	0.2050	449.213	54.085	0.9604	1209.220	246.153	**0.0212**	1581.167	42.413	0.6440	10.259	8.171	0.2943

AVG: average; SD: standard deviation; *p*-value: probability value.

**Table 3:** The effect of CAPE on the production of selected cytokines IL-1β, IL-6, IL-8, IL-15, TNF-α and CAPE in LPS, EEP in IFN-α, as well as CAPE in LPS + IFN-α-stimulated HGF-1 cells (*n* = 3). Statistical significance was analysed using Fisher’s LSD test. Results marked in bold are statistically significant in Fisher’s LSD test. Multivariate tests of significance (F = 12.91, *p* < 0.05).

Sample	IL-1β			IL-6			IL-8			IL-15			TNF-α		
	AVG	SD	*p*	AVG	SD	*p*	AVG	SD	*p*	AVG	SD	*p*	AVG	SD	*p*
Control baseline (CAPE)	2.440			7.020			1.420			97.040			7.779		
Control DMSO (CI)	1.988	0.157		315.380	99.899		37.013	10.225		150.359	5.142		6.741	1.372	
Control LPS (CLI)	4.616	4.247		64,261.308	28,492.806		6603.190	1208.399		230.669	148.971		23.070	12.780	
Control IFN-α (CI)	2.079	0.157		446.373	41.877		17.213	6.016		211.100	94.533		5.877	0.599	
Control LPS + IFN-α (CLI)	5.016	2.708		91,701.724	26,094.008		6392.827	913.894		177.278	39.430		43.047	22.899	
CAPE 25 µg/mL	2.079	0.157		310.367	25.022		40.087	1.184		140.821	7.954		6.223	1.037	
CAPE 50 µg/mL	1.717	0.313		338.890	33.819		56.207	10.582		152.042	8.637		6.050	0.792	
CAPE 25 µg/mL + LPS	2.350	0.157	0.0846	6551.583	418.252	**0.0000**	6002.223	1171.625	0.2323	223.604	120.550	0.9443	12.063	4.726	0.0938
CAPE 50 µg/mL + LPS	2.395	0.078	0.0909	6175.093	940.004	**0.0000**	7580.387	1974.254	0.0548	153.725	3.887	0.4480	16.351	13.503	0.3022
CAPE 25 µg/mL + IFN-α	1.988	0.157	0.5182	582.057	72.812	0.9827	28.070	11.323	0.9827	135.772	8.303	0.4575	7.260	1.037	0.8310
CAPE 50 µg/mL + IFN-α	1.807	0.564	0.4960	565.647	36.875	0.9848	35.733	14.639	0.9704	137.455	16.173	0.4676	6.914	0.599	0.8729
CAPE 25 µg/mL + LPS + IFN-α	4.056	3.037	0.4598	11,247.297	2229.940	**0.0000**	6558.080	384.514	0.7410	150.359	5.142	0.7902	23.873	17.071	**0.0045**
CAPE 50 µg/mL + LPS + IFN-α	3.876	3.194	0.3803	14,107.183	2445.806	**0.0000**	8240.420	1333.419	**0.0005**	344.700	85.699	0.1022	24.301	13.609	**0.0054**

## Data Availability

Data are contained within the article and Supplementary Materials.

## Supplementary Materials

The following supporting information can be downloaded at: https://www.mdpi.com/article/10.3390/molecules28227477/s1.
