# Supplementary material for: The Phenolic Profile and Anti-Inflammatory Effect of Ethanolic Extract of Polish Propolis on Activated Human Gingival Fibroblasts-1 Cell Line
Source: Molecules. 2023 Nov 8;28(22):7477. doi: 10.3390/molecules28227477 (PMC10673102; doi:10.3390/molecules28227477)
Supplement: Supplementary file 1 [file molecules-28-07477-s001.zip › molecules-2607210-supplementary.pdf]

molecules

MDPI

Supplementary Materials

The Phenolic Profile and Anti-Inflammatory Effect of Ethanolic Extract of Polish Propolis on Activated Human Gingival Fibroblasts-1 Cell Line

Anna Kurek-Górecka [1,*], Małgorzata Kłósek [2], Grażyna Pietsz [2], Zenon P. Czuba [2,*], Sevgi Kolayli [3], Zehra Can [4], Radosław Balwierz [5] and Paweł Olczyk [1]

[1] Department of Community Pharmacy, Faculty of Pharmaceutical Sciences in Sosnowiec, Medical University of Silesia in Katowice, Kasztanowa 3, 41-200 Sosnowiec, Poland; polczyk@sum.edu.pl
[2] Department of Microbiology and Immunology, Faculty of Medical Sciences in Zabrze, Medical University of Silesia in Katowice, Jordana 19, 41-808 Zabrze, Poland; mklosek@sum.edu.pl (M.K.); gpietsz@sum.edu.pl (G.P.)
[3] Department of Chemistry, Faculty of Science, Karadeniz Technique University, 61080 Trabzon, Turkey; skolayli61@yahoo.com
[4] Department of Emergency Aid and Disaster Management, Faculty of Applied Sciences, Bayburt University, 69000 Bayburt, Turkey; zehracan61@gmail.com
[5] Institute of Chemistry, University of Opole, Oleska 48, 45-052 Opole, Poland; radoslaw.balwierz@uni.opole.pl
* Correspondence: akurekgorecka@sum.edu.pl (A.K.-G.); zczuba@sum.edu.pl (Z.P.C.)

Table of content	page
**Table S1.** Chemical structures of phenolic compounds detected in propolis extracts (Table S1).	2
**Table S2.** LOD and LOQ values of standards (Table S2).	3
**Table S3**. Average, standard deviations, statistical significance of cytotoxic activity of EEP (Table S3).	4
**Table S4.** Average, standard deviations, statistical significance of cytotoxic activity of CAPE (Table S4).	5
**Figure S1.** HGF-1 cells (magnification 20x) before incubation with MTT test. (The arrow marks HGF-1).	6
**Figure S2.** HGF-1 cells incubated with MTT for 4 hours presenting formazan crystals. (The arrow mark the selected formazan crystals).	6
**Figure S3.** Flowchart of experimental study.	7

Table S1. Chemical structures of phenolic compounds detected in propolis extracts (Table S1).

Phenolic compound	Structure
gallic acid	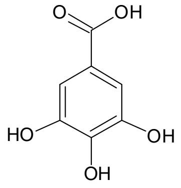
chlorogenic acid	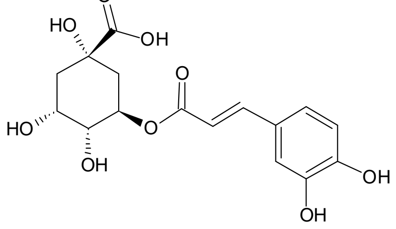
caffeic acid	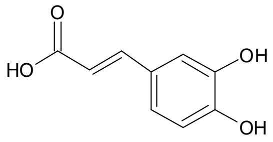
p-coumaric acid	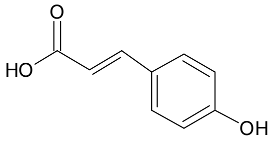
ferulic acid	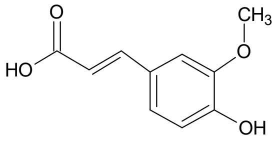
luteolin	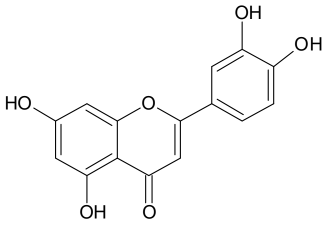
quercetin	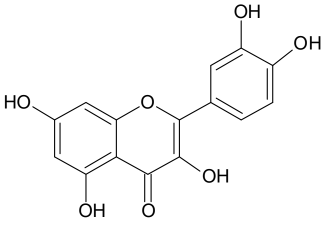
t-cinnamic acid	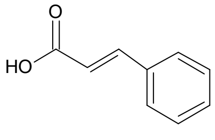
apigenin	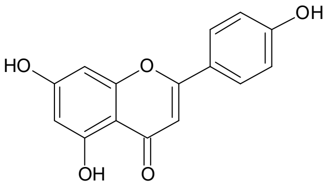

chrysin	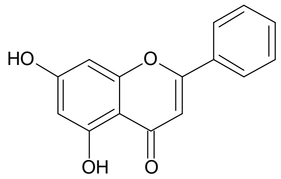
pinocembrin	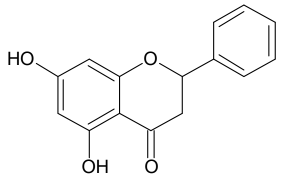
caffeic acid phenethyl ester (CAPE)	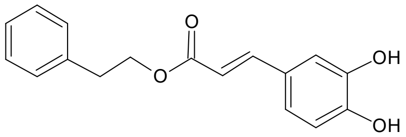

Table S2. LOD and LOQ values of standards.

Standards	Limit of Detection (LOD) (μg/mL)	Limit of Quantification (LOQ) (μg/mL)
gallic acid	0.0099	0.0331
protocatechuic acid	0.0042	0.0139
chlorogenic acid	0.0199	0.0662
p-OH benzoic acid	0.0309	0.1031
epicatechin	0.0569	0.1896
caffeic acid	0.0859	0.2865
syringic acid	0.0203	0.0676
m-OH benzoic acid	0.0074	0.0247
rutin	0.0838	0.2793
ellagic acid	0.0896	0.2988
p-coumaric acid	0.0333	0.1108
ferulic acid	0.0196	0.0653
myricetin	0.0868	0.2895
resveratrol	0.0336	0.1120
daidzein	0.0230	0.0768
luteolin	0.0254	0.0847
quercetin	0.0022	0.0074
t-Cinnamic acid	0.0286	0.0954
apigenin	0.0439	0.1463
hesperidin	0.0035	0.0117
rhamnetin	0.0165	0.0546
chrysin	0.0206	0.0687
pinocembrin	0.0852	0.2841
CAPE	0.0037	0.0124
curcumin	0.0908	0.3027

Table S3. Average, standard deviations, statistical significance of cytotoxic activity of EEP. Statistical significance was calculated using t-test (Table S2).

Sample	HGF-1 cell viability [%]		
	AVG	SD	*p*
Control base line	92.84	4.16	
EEP 10 μg/mL	120.65	9.73	0.000
EEP 25 μg/mL	121.38	6.48	0.000
EEP 50 μg/mL	129.48	7.74	0.000
EEP 100 μg/mL	128.67	11.08	0.000
Control IFN-α	94.84	9.92	
EEP 10 μg/mL + IFN-α	106.20	5.47	0.001
EEP 25 μg/mL + IFN-α	100.41	4.90	0.036
EEP 50 μg/mL + IFN-α	97.50	6.27	0.354
EEP 100 μg/mL + IFN-α	102.82	8.71	0.044
Control LPS	90.42	5.89	
EEP 10 μg/mL + LPS	120.72	15.55	0.001
EEP 25 μg/mL + LPS	104.42	6.90	0.001
EEP 50 μg/mL + LPS	124.62	4.76	0.000
EEP 100 μg/mL + LPS	117.36	9.75	0.000
Control LPS + IFN-α	91.32	7.66	
EEP 10 μg/mL + LPS + IFN-α	123.27	2.33	0.000
EEP 25 μg/mL + LPS + IFN-α	115.67	1.68	0.000
EEP 50 μg/mL + LPS + IFN-α	120.08	2.84	0.000
EEP 100 μg/mL + LPS + IFN-α	113.93	2.09	0.000

Table S4. Average, standard deviations, and statistical significance of cytotoxic activity of CAPE. Statistical significance was calculated using t-test (Table S3).

Sample	HGF-1 cell viability [%]		
	AVG	SD	*p*
Control base line	95.84	6.94	
CAPE 10 µg/mL	106.64	12.97	0.032
CAPE 25 µg/mL	127.30	10.79	0.000
CAPE 50 µg/mL	138.58	12.74	0.000
CAPE 100 µg/mL	154.53	7.67	0.000
Control IFN-α	88.32	6.82	
CAPE 10 µg/mL + IFN-α	128.21	2.78	0.000
CAPE 25 µg/mL + IFN-α	127.82	3.29	0.000
CAPE 50 µg/mL + IFN-α	135.13	4.69	0.000
CAPE 100 µg/mL + IFN-α	144.07	4.52	0.000
Control LPS	93.62	4.88	
CAPE 10 µg/mL + LPS	138.85	2.47	0.000
CAPE 25 µg/mL + LPS	140.58	5.51	0.000
CAPE 50 µg/mL + LPS	145.73	5.51	0.000
CAPE 100 µg/mL + LPS	158.16	5.09	0.000
Control LPS + IFN-α	97.55	0.10	
CAPE 10 µg/mL + LPS + IFN-α	104.62	2.57	0.000
CAPE 25 µg/mL + LPS + IFN-α	102.08	2.08	0.000
CAPE 50 µg/mL + LPS + IFN-α	99.23	9.50	0.638
CAPE 100 µg/mL + LPS + IFN-α	101.46	10.29	0.323

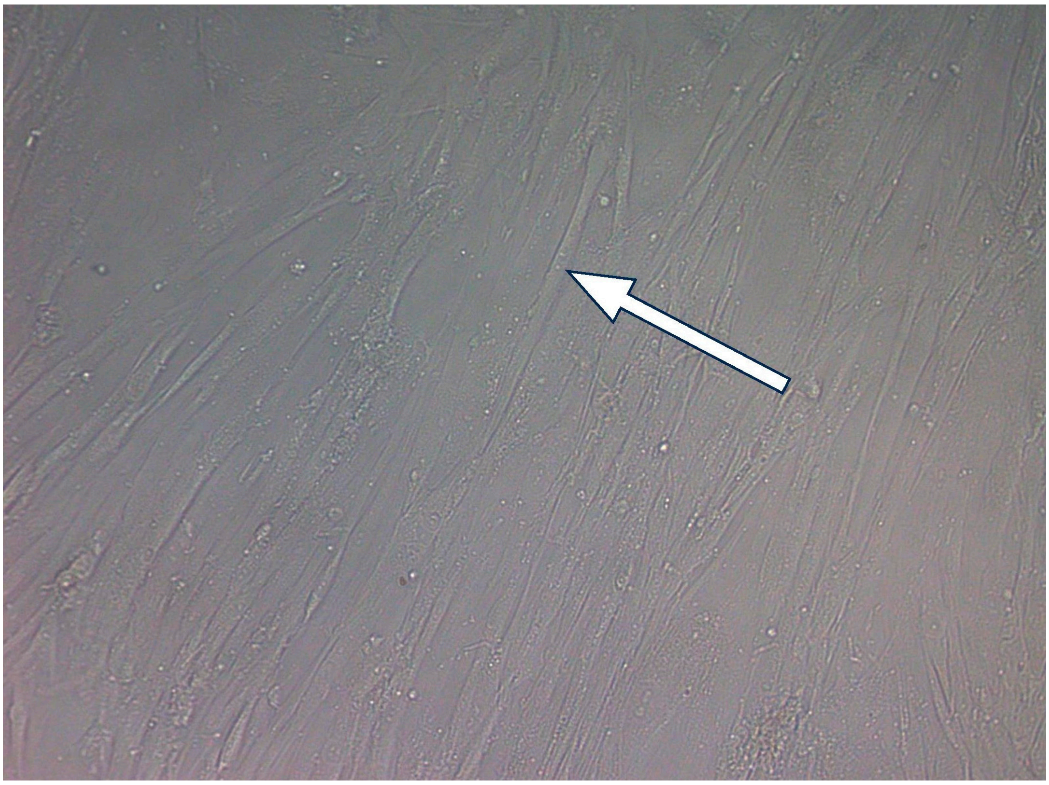

Figure S1. HGF-1 cells (magnification 20x) before incubation with MTT test. (The arrow marks HGF-1).

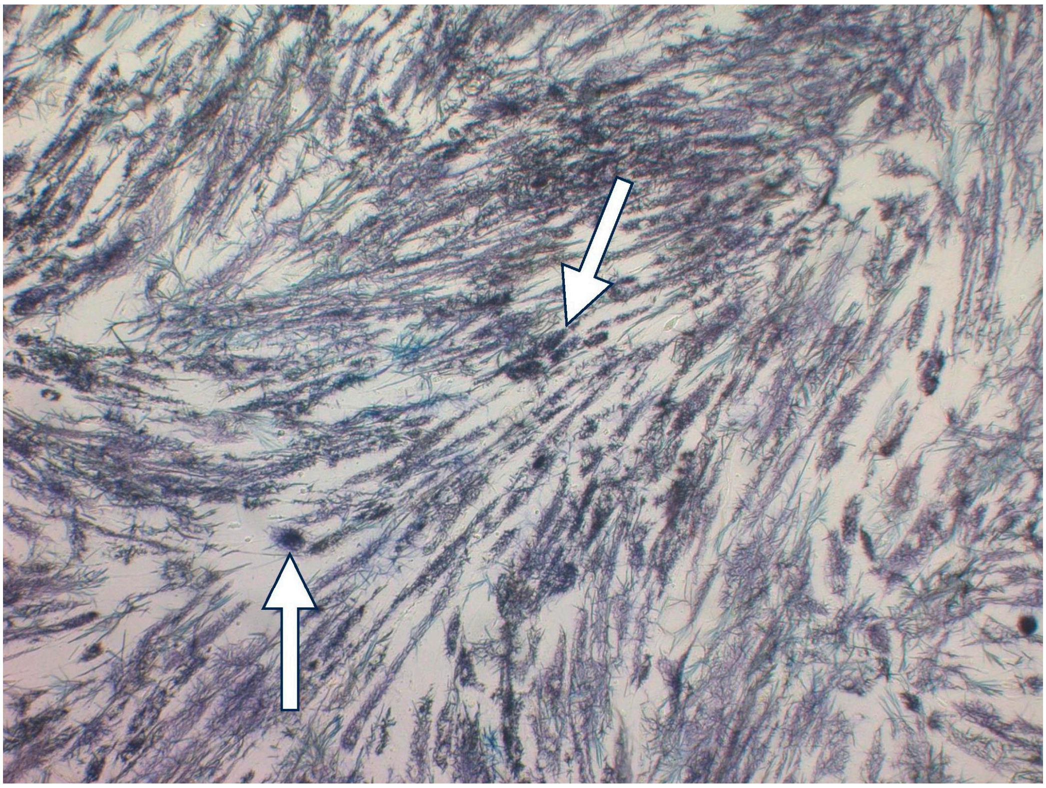

Figure S2. HGF-1 cells incubated with MTT for 4 hours presenting formazan crystals. (The arrow mark the selected formazan crystals).

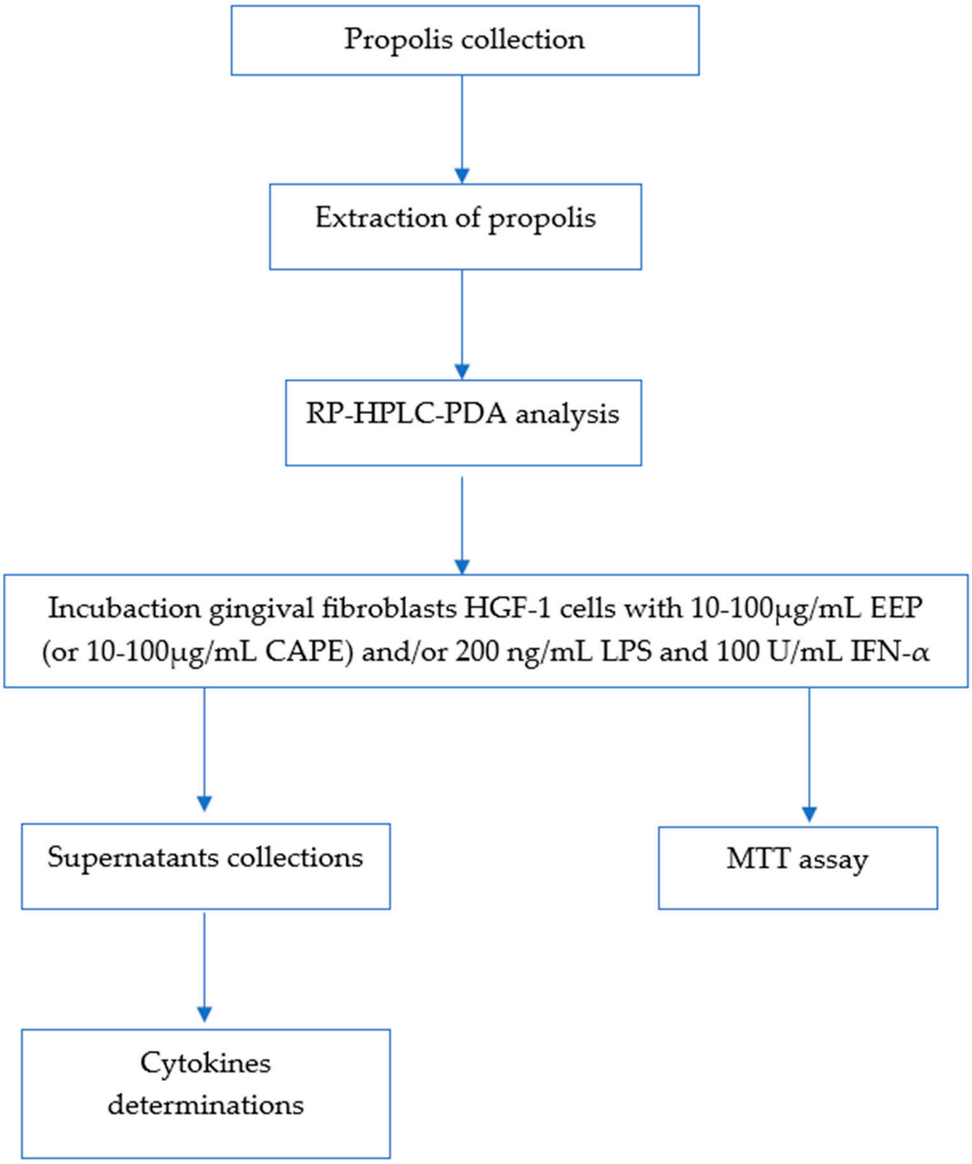

Figure S3. Flowchart of experimental study.
